# Supplementary material for: The association between neutrophil and lymphocyte to high-density lipoprotein cholesterol ratio and metabolic syndrome among Iranian population, finding from Bandare Kong cohort study
Source: Lipids Health Dis. 2024 Nov 28;23:393. doi: 10.1186/s12944-024-02378-5 (PMC11603836; doi:10.1186/s12944-024-02378-5)
Supplement: Supplementary file 1 — Supplementary Material 1 [file 12944_2024_2378_MOESM1_ESM.pdf]

ithenticate_40084494.pdf

WORD COUNT	5673	TIME SUBMITTED	15-NOV-2024 07:28AM
		PAPER ID	112961781

Seyyed Mohammad Hashemi[1,2], Masoumeh Kheirandish[3], Shideh Rafati[4], Arezoo Ghazalgoo[1], Ehsan Amini-Salehi[5], Mohammad-Hossein Keivanlou[6], Shahin Abbaszadeh[2], Parsa Saberian[1,2*], Arash Rahimi[3*]

***Corresponding Author**

Arash Rahimi

arashrahimi.article@gmail.com

***Co-Corresponding Author:**

Parsa Saberian

Abstract

Background: Metabolic Syndrome (MetS) is characterized by the co-occurrence of various metabolic risk factors, significantly increasing the risk of cardiovascular diseases (CVD) and type 2 diabetes (T2DM). This study investigates the potential of hematological indices, specifically the neutrophil to high-density lipoprotein cholesterol ratio (NHR) and lymphocyte to high-density lipoprotein cholesterol ratio (LHR), as predictors of MetS in a population from southern Iran.

Methods: Utilizing baseline data from the Bandare-Kong Non-Communicable Diseases (BKNCD) Cohort, part of the Prospective Epidemiological Research Studies in IrAN (PERSIAN), A total of 2,684 participants aged 35–70 years were analyzed. Participants were evaluated using the Iranian National Cholesterol Education Program (NCEP) criteria to diagnose MetS. Receiver operating characteristic (ROC) analysis was conducted to assess the predictive validity of NHR and LHR across different demographic categories.

Results: The mean LHR and NHR values were significantly higher in individuals diagnosed with MetS ($P < 0.001$). Specifically, the LHR was 0.85 ± 0.26 in MetS patients compared to 0.76 ± 0.23 in those without MetS, while the NHR was 1.33 ± 0.35 in MetS patients compared to 1.20 ± 0.32 in those without MetS. After adjusting for confounding factors, both LHR and NHR remained significantly associated with MetS, with odds ratios (OR) of 6.61 (95% CI: 4.43-9.83) for LHR and 4.76 (95% CI: 3.51-6.45) for NHR. Among MetS components, LHR was associated with low HDL cholesterol and elevated triglycerides, while NHR showed significant associations with central obesity, low HDL cholesterol, and elevated triglycerides. ROC analysis revealed moderate predictive capabilities for both indices, with areas under the curve of 0.60 for LHR and 0.61 for NHR.

Conclusion: The findings suggest that NHR and LHR are promising, easily obtainable hematological markers for predicting MetS. These indices could serve as valuable tools for early detection and ongoing monitoring in clinical settings, aiding in the prevention and management of MetS.

Introduction

MetS presents a significant global health challenge, encompassing a range of interrelated metabolic disorders including systemic hypertension, insulin resistance, central obesity, and atherogenic dyslipidemia [1]. Epidemiological data consistently demonstrate that individuals with MetS face substantially higher risks of serious health conditions, including a five-fold increase in the likelihood of developing T2DM and a two-fold higher risk of CVD [2, 3]. Prevalence rates of MetS are strikingly high, with national studies reporting rates between 32% and 47.6%, while systematic reviews estimate that approximately 30.4% of the Iranian population is affected [4, 5]. Additionally, data from the BKNCD cohort study in southern Iran indicate that 34.5% of individuals in this region are living with MetS [6].

The pathophysiology of MetS is complex, involving genetic, environmental, and metabolic factors[7-9]. Central to MetS is insulin resistance, often driven by visceral obesity, which promotes a pro-inflammatory state and endothelial dysfunction[10-14]. Dyslipidemia, characterized by high triglyceride (TG) and low HDL-C levels, further contributes to MetS. Chronic inflammation and oxidative stress are also key players[15-17]. Emerging evidence highlights the role of gut microbiota in MetS, with alterations in microbial composition influencing energy metabolism, fat

storage, and inflammation, thereby contributing to the development of MetS and related metabolic disorders[18-22].

Research into the inflammatory processes associated with MetS has produced a wide range of findings, particularly concerning hematological parameters such as red blood cells, platelets, white blood cells , and various inflammatory markers [23-25]. These variations underscore the complexity of MetS and the difficulty in pinpointing consistent biomarkers.

For instance, some studies have documented a decrease in lymphocyte count among MetS patients [26, 27], suggesting a potential immune suppression aspect. In contrast, other research indicates a possible connection between lymphocyte proliferation and the onset of MetS [28, 29]. Additionally, under normal physiological conditions, HDL-C plays a protective role by inhibiting macrophage migration and fat deposition in blood vessel walls [27]. However, in the context of MetS, the reduction in HDL-C levels may accelerate the development of atherosclerosis, thereby worsening cardiovascular outcomes.

Numerous studies have established correlations between peripheral blood counts and metabolic components of MetS [30-32]. However, the limitations of using individual hematological parameters as reliable predictors for MetS have prompted the exploration of such indices, such as NHR and LHR, which may offer more accurate assessments. This study hypothesizes that NHR and LHR, as composite hematological indices, provide a more robust predictive and diagnostic approach for MetS than individual blood parameters due to their combined reflection of inflammatory and lipid profiles. By examining these associations, this research seeks to contribute valuable insights to the existing knowledge on MetS biomarkers, supporting the development of enhanced diagnostic tools and therapeutic strategies. Such advancements are crucial to meeting

the increasing demand for early detection and effective management of MetS, both within Iran and on a global scale.

Method

Study design and sampling

A cross-sectional population-based study was conducted using baseline data from the BKNCD Cohort Study, a segment of the PERSIAN.The PERSIAN cohort study enrolled individuals aged 35-70 from 18 distinct geographical regions across Iran, with detailed information available[33] . Initially, the study encompassed 4,063 participants aged 35–70, recruited between November 2016 and November 2018 from Bandare-Kong, Hormozgan Province, in southern Iran.

Exclusion criteria were applied to pregnant women, incomplete or insufficient data, and patients with conditions capable of impacting the blood system, including acute or ongoing infections, end-stage renal or liver diseases, cancers, hematologic malignancies, CVD, and those on antihyperlipidemic or corticosteroid treatment. By refining the study sample through these exclusions, potential confounding variables were minimized, enhancing the validity of the results. Following these exclusions, the final analysis included a total of 2,684 participants (1,328 males and 1,356 females), ensuring a balanced representation. The study sample was further divided into 802 cases and 1,882 controls.

Data Collection

Key demographic information, such as age, gender, educational background, marital status, occupation, and social factors like smoking habits, dietary patterns, and levels of physical activity, was collected. Participants' daily food consumption was documented using a food frequency questionnaire (FFQ), and daily calorie intake was calculated based on the caloric content of the consumed items. Trained personnel conducted face-to-face interviews using validated and reliable questionnaires, specifically designed for consistent data collection across all sites in the PERSIAN cohort.

Anthropometric and Biochemical Measurements

A digital scale was carefully calibrated and used to measure the weight of each participant, ensuring precision by instructing participants to wear only light clothing and no shoes. This method minimizes measurement variability and helps standardize weight data across the study. Each weight was rounded to the nearest 0.5 kg to maintain uniformity in data recording and analysis. Height was measured using a stadiometer, a precise instrument specifically designed for height assessment. Participants were instructed to stand straight with their feet flat on the ground, shoulders relaxed, and head positioned in the Frankfort horizontal plane to ensure accuracy. By removing shoes, any discrepancies due to footwear were eliminated, thereby enhancing the reliability of the height data.

Waist circumference (WC) measurements were obtained following specific anatomical landmarks to ensure consistency. The measurement site was set at the midpoint between the top of the iliac crest and the lower edge of the last palpable rib along the mid-axillary line. This location provides an accurate representation of central adiposity. Each WC measurement was performed twice by

trained professionals using a non-stretch tape, and the average of these readings, rounded to the nearest 0.5 cm, was recorded to ensure accuracy and reduce potential measurement error. Body Mass Index (BMI) was calculated as weight in kilograms divided by height in meters squared. This index serves as an indicator of overall body fat distribution and is a commonly used metric for categorizing weight status.

Blood pressure (BP) assessments included both systolic (SBP) and diastolic (DBP) readings using a standard mercury sphygmomanometer. Participants were instructed to sit comfortably with their arms at heart level, and they were given a 15-minute rest period prior to measurement to eliminate any temporary influence of stress or physical exertion. Each participant's SBP and DBP were measured twice, and the mean of these measurements was recorded for precision.

For biochemical analysis, fasting blood samples were collected after participants had fasted for at least 12 hours overnight. Fasting plasma glucose (FPG) levels were determined using the glucose oxidase method, which is known for its high specificity in glucose detection. Lipid profile measurements—including total cholesterol (TC), TG, low-density lipoprotein (LDL), and high-density lipoprotein cholesterol (HDL-C)—were conducted using the enzymatic method, which provides reliable and reproducible results for lipid quantification, essential for assessing cardiovascular risk factors.

The criteria established by the Iranian National Committee of Obesity were utilized to identify MetS, with any three out of five criteria met indicating a person's qualification for MetS [34]:

1. **WC**: A waist measurement of 95 cm or more. This threshold indicates central obesity, which is a significant risk factor for MetS.

2. **FPG**: A blood glucose level of 100 mg/dL or higher, or the individual is currently receiving medical treatment to control elevated blood sugar. High fasting glucose can be an indicator of insulin resistance, a key component of MetS.
3. **HDL Cholesterol Levels**: For men, HDL levels should be below 40 mg/dL, while for women, they should be below 50 mg/dL. Alternatively, if the individual is undergoing treatment for low HDL levels, they also meet this criterion. Low HDL is considered a risk factor because HDL helps remove cholesterol from the bloodstream.
4. **TG Levels**: Triglyceride levels of 150 mg/dL or higher, or current treatment for elevated triglycerides, also indicate a risk. Elevated triglycerides are associated with a higher likelihood of heart disease and other MetS-related conditions.
5. **BP:** BP readings of 130/85 mmHg or higher, or ongoing treatment for hypertension, are also a marker. High BP places additional strain on the heart and blood vessels, increasing the risk of cardiovascular issues.

Meeting any three of these five criteria is typically considered enough to diagnose MetS. Each of these factors contributes to an increased risk of developing CVD, diabetes, and other health complications, highlighting the importance of regular health screenings and preventive measures.

Data analysis

This research constitutes a descriptive, cross-sectional analysis of data obtained from the Bandar Kong cohort study, aimed at identifying patterns and relationships related to MetS among participants. In this analysis, categorical variables were represented using frequencies and percentages (%), enabling an overview of the distribution of these variables within the sample population. Continuous variables were summarized through mean values and standard deviations

(SD) to illustrate central tendencies and the variability within the data. To assess differences in the average values of continuous variables between two groups, the t-test was employed, providing insight into statistically significant mean differences. For categorical data, the chi-square test was used to evaluate the association between two categorical variables, offering a measure of whether relationships observed within the data were likely to have occurred by chance.

Further analysis involved logistic regression to explore the potential relationship between two critical ratios—NHR and LHR—and their association with MetS. This method helped quantify the likelihood of MetS in relation to changes in these ratios. To determine the predictive power of NHR and LHR ratios in identifying MetS and its components, the ROC curve and the Area Under the Curve (AUC) were employed. These metrics provide an assessment of how well the NHR and LHR ratios can differentiate between individuals with and without MetS, with the AUC offering a numerical measure of predictive accuracy. To enhance the precision of the diagnostic tool, Youden's J statistic was applied to calculate the optimal cut-off values for NHR and LHR. This statistic was used to maximize the combined sensitivity and specificity (sensitivity + specificity - 1) for detecting MetS, ultimately improving the practical utility of these ratios as screening markers.

The ROC curve analysis was conducted using MedCalc Version 20, a software known for its robust tools in medical statistics, while all other statistical procedures, including general data analysis and validation, were performed using IBM SPSS Version 25 to ensure consistency and accuracy across different analytical methods. A two-sided *P*-value of less than 0.05 was considered indicative of statistical significance, ensuring that findings were robust and unlikely to be due to random variation alone.

Results

Among the 2684 participants, 29.9% (802 individuals) were afflicted by MetS. Results revealed significant association between various demographic factors—such as age, gender, marital status, place of residence, education, employment status, and physical activity—and the presence of MetS ($P<0.05$). As age advanced, there was a corresponding escalation in the prevalence of MetS, with women exhibiting a higher prevalence (33.7%) compared to men (26.0%). Those who were divorced or widowed presented a notably higher prevalence of MetS (40.4%) compared to their single or married counterparts. Moreover, individuals residing in urban areas, possessing lower educational attainment, experiencing unemployment, and engaging in minimal physical activity demonstrated the highest prevalence of MetS. These baseline demographic characteristics of the population are presented in Table 1.

The results revealed significant differences between individuals with MetS and those without it in various variables (Table 2). Participants with MetS had an average age of 50.35 ± 9.23 years, which was significantly higher than the 46.11 ± 8.73 years observed in those without MetS ($P < 0.01$). MetS group exhibited a significantly higher BMI, with a mean of 29.16 ± 4.23, compared to a mean of 25.16 ± 4.43 in the non-MetS group ($P < 0.01$). Regarding hematological indices, the NHR and the LHR were both elevated in the MetS group. The NHR was 1.33 ± 0.35 in MetS patients, compared to 1.20 ± 0.32 in those without MetS ($P < 0.01$). Similarly, the LHR was higher in the MetS group, with a mean of 0.85 ± 0.26 versus 0.76 ± 0.23 in the non-MetS group ($P < 0.01$).

The results of the binary logistic regression models indicated that both NHR and LHR are significant risk factors for MetS, after adjusting for other variables such as age, BMI, gender, and physical activity.

For NHR, the adjusted odds ratio (OR) for MetS was 4.76 (95% CI: 3.51, 6.45 (Table 3). Similarly, for LHR, the adjusted OR was 6.61 (95% CI: 4.43, 9.83) (Table 4).

Additionally, age was found to be a significant predictor in both models, with each year increase in age associated with an adjusted OR of 1.08 (95% CI: 1.07, 1.09) in the NHR model and 1.07 (95% CI: 1.06, 1.09) in the LHR model (Tables 3 and 4).

BMI also demonstrated a significant association with MetS, with an adjusted OR of 1.25 (95% CI: 1.22, 1.28) in the NHR model and 1.26 (95% CI: 1.23, 1.29) in the LHR model (Tables 3 and 4).

Gender differences were noted, with females having a higher risk of MetS compared to males, as reflected by an adjusted OR of 1.24 (95% CI: 1.01, 1.52) in the NHR model and 1.20 (95% CI: 0.98, 1.47) in the LHR model (Tables 3 and 4).

Physical activity was inversely related to MetS, with moderate levels of physical activity (36.6-44.9 METS/week) showing a protective effect against MetS, as indicated by an adjusted OR of 1.46 (95% CI: 1.08, 1.98) in the NHR model and 1.37 (95% CI: 1.02, 1.84) in the LHR model (Tables 3 and 4).

Based on the findings presented in Table 5, significant differences in the NHR were observed in relation to central (abdominal) obesity, triglyceride levels, and HDL cholesterol levels. Specifically, individuals with high abdominal obesity had a significantly higher NHR (1.27 ± 0.33) compared to those with normal abdominal measurements (1.22 ± 0.33, $P < 0.01$). Additionally, those with elevated triglycerides and low HDL cholesterol levels also exhibited significantly higher NHR values (1.31 ± 0.35 vs. 1.20 ± 0.32, $P < 0.01$; and 1.47 ± 0.32 vs. 1.09 ± 0.25, $P < 0.01$, respectively). Similarly, LHR showed significant associations with triglyceride levels and HDL cholesterol levels. Participants with high triglyceride levels had a higher LHR (0.86 ± 0.26) compared to those with normal levels (0.76 ± 0.23). Furthermore, those with low HDL cholesterol had a higher LHR (0.93 ± 0.26) compared to individuals with normal HDL levels (0.70 ± 0.19). No significant differences in NHR or LHR were found in relation to hypertension or impaired fasting glucose (IFG).

According to the ROC analysis, as illustrated in Figure 1 and summarized in Table 6, the results indicated that both NHR and LHR performed similarly in predicting diagnosed MetS for both sexes, with AUC values slightly above 0.6. These findings suggested that both NHR and LHR could be considered moderate predictors for identifying MetS in both men and women. The optimal cut-off points for NHR and LHR were determined using Youden's J statistic. For NHR, the optimal cut-off values for screening MetS were identified as 1.29 for men and 1.13 for women. In the case of LHR, the optimal cut-off values were 0.91 for men and 0.68 for women (Table 6). Table 7 presented the association between actual MetS and MetS predicted by logistic regression models, which were adjusted for age, BMI, and physical activity for both sexes. The analysis showed that when NHR was used as a predictor, the association between MetS and predicted MetS was statistically significant for both men and women ($P < 0.05$). However, when LHR was used as a predictor, the association was statistically significant only in men ($P < 0.05$), and not in women. The strength of the association between actual and predicted MetS was assessed using Somers' d, Kendall's tau, and Goodman and Kruskal's gamma indices.

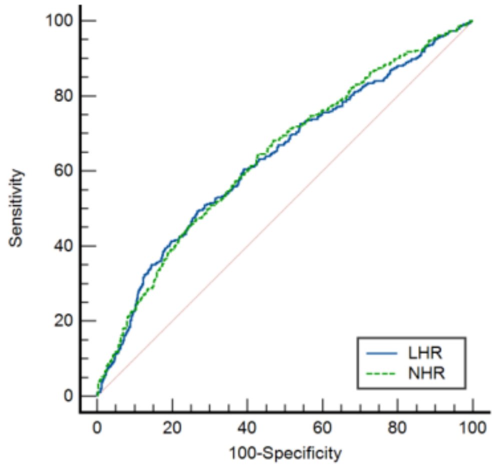
Sensitivity
100
80
60
40
20
0
0
20
40
60
80
100
100-Specificity
LHR
NHR

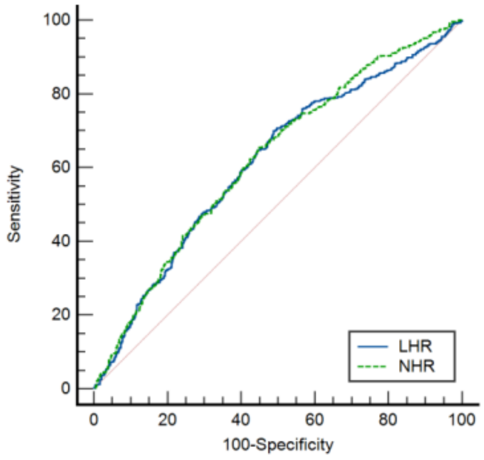
Sensitivity
100
80
60
40
20
0
0
20
40
60
80
100
100-Specificity
LHR
NHR

Discussion

In this cross-sectional study, the neutrophil and lymphocyte to HDL cholesterol ratios were evaluated to assess their potential contribution to the diagnosis and prediction of MetS occurrence in a large population in southern Iran. MetS is identified by a set of risk factors, including insulin resistance, which is associated with subclinical chronic inflammation [35]. Blood parameters can be used as indirect predictors of this inflammation. Therefore, changes in blood parameters have important prognostic implications for chronic inflammation and the progression of MetS.

Many investigations have shown changes in hematological parameters in high-risk patients for MetS. Wang et al. discovered that individuals in the highest quartile of WBC counts had three times the risk of MetS compared to those in the lowest quartile [36]. Nagasawa et al. conducted research including 3,594 Japanese males between the ages of 34 and 69. They discovered that there was a positive correlation between WBC count and BMI, BP, triglycerides, glucose, and insulin levels. Conversely, there was a negative correlation between WBC count and HDL cholesterol. Individuals diagnosed with MetS had elevated WBC counts and insulin levels. [37]. Consistent

with this study, a cross-sectional survey among 1,401 adults in China demonstrated that LHR and NHR are strong predictors of MetS in females, independent of other factors. In males, while LHR and NHR were initially significant predictors, their significance diminished after adjusting for fasting glucose levels [23]. ROC analysis indicated that LHR is the best predictor for MetS in females and NHR in males. In the present study, LHR and NHR were significantly higher in MetS patients (<0.001). In addition, there was a significant association between LHR and NHR and the incidence of MetS after adjusting for age, gender, BMI, smoking, economic state, and activity.

In line with this study, an investigation in China revealed a positive correlation between MetS and blood parameters. ROC analysis showed that LHR is an independent predictor of MetS with a cut-off value of 1.657, sensitivity of 65%, and specificity of 64% [38]. Assessing the accuracy of LHR and NHR with ROC curve analysis in this study indicated that NHR and LHR had the same acceptable value for Mets (AUC; LHR: %60, NHR: %61).

In another study conducted by Najafzadeh et al. [39] on a southeastern Iranian population, it was found that the NHR was significantly associated with the severity of MetS. These findings align with these results, further highlighting the utility of NHR as a predictive marker for MetS across different Iranian populations.

Another study in rural China found that the LHR can effectively predict newly diagnosed MetS. The study also discovered that individuals with higher LHR values tended to be younger, male, and smokers. Additionally, they had a higher WC, higher levels of triglycerides, and lower levels of HDL. The study concluded that LHR could be useful for identifying rural individuals at risk of MetS as a new inflammatory marker. However, the platelet-to-lymphocyte ratio (PLR) was insignificant in predicting MetS [24]. The associations between the NHR and LHR with various components of MetS were investigated. The comprehensive study included both urban and rural

populations, and the findings were closely aligned with previous research. Analysis indicated that NHR and LHR had the strongest correlations with elevated TG levels and reduced HDL cholesterol levels. Moreover, NHR demonstrated a significant association with central obesity, underscoring its relevance as an indicator of this particular aspect of MetS. These results suggest that NHR and LHR are valuable markers for identifying specific metabolic abnormalities across diverse population settings.

In another study, researchers found that in patients with early-stage MetS, the ratios of leukocytes (specifically PMNs and monocytes) to HDL-C and adiponectin were significantly higher [40]. Additionally, they discovered that the ratios of PMN to HDL-C and monocyte to HDL-C were better indicators of MetS compared to high-sensitivity C-reactive protein (hs-CRP). WBC count is a commonly used marker for mild systemic inflammation. However, high-sensitivity hs-CRP has been identified as a superior marker for evaluating the inflammatory aspects of MetS. Numerous studies have linked hs-CRP with diabetes, CVD , and MetS [30-32, 41]. Despite this, WBC counts may be more advantageous in clinical settings due to their affordability and ease of access, simplifying diagnosis and treatment while reducing costs [42, 43].

Furthermore, in various studies where CRP has been utilized as an inflammatory marker for diagnosing MetS in comparison to NHR and LHR, its application has often been accompanied by notable limitations. For instance, according to Chen et al. [23], CRP is a less effective predictor of MetS due to its susceptibility to confounding factors such as infections and tissue injuries, which can lead to elevated levels that are unrelated to the syndrome. In contrast, NHR and LHR are more closely linked to chronic inflammation, a key driver of MetS, and maintain their predictive accuracy even after adjusting for factors like fasting glucose. Moreover, CRP is a general

inflammatory marker, while NHR and LHR are more specifically associated with inflammation linked to MetS, making them more reliable indicators [23].

Additionally, the latest systematic review by Podeanu et al. [44] underscores several limitations in using CRP as a marker for MetS. One major limitation is its inability to distinguish between patients with MetS and those who are simply obese, as elevated CRP levels may be a result of obesity-related inflammation rather than the syndrome itself. Moreover, variability in measurement methods, such as standard CRP versus hsCRP, can influence diagnostic accuracy. CRP levels are also influenced by various confounding factors, such as infections and tissue damage, further complicating the diagnosis of MetS [44].

Also, Marra et al. [45] demonstrated that the NHR and LHR showed clear superiority over other examined inflammatory markers in individuals with MetS. These two ratios outperformed other CBC-derived indices, such as monocyte-to-HDL-C ratio, platelet-to-HDL-C ratio, SIRI (Systemic Inflammation Response Index), SII (Systemic Immune-Inflammation Index), and AISI (Aggregate Index of Systemic Inflammation), in predicting the severity of MetS. Moreover, NHR and LHR were significantly more associated with cardiometabolic risk factors such as HOMA-IR and the TG/HDL-C ratio, further highlighting their stronger predictive power. While SIRI also showed some correlation with these factors, SII and AISI demonstrated weaker associations and lower predictive value [45].

The mechanisms underlying this association are not yet fully understood, though several plausible hypotheses have been proposed [46] . MetS is characterized by a combination of factors that are closely linked to chronic low-grade inflammation. Elevated neutrophil levels in individuals with

MetS serve as markers of increased systemic inflammation, driven in part by pro-inflammatory cytokines such as interleukin-6 (IL-6) and tumor necrosis factor-alpha (TNF-α). These cytokines induce oxidative stress, which in turn worsens insulin resistance—one of the key hallmarks of MetS. Neutrophils, as part of the innate immune system, proliferate in response to these cytokines, indicating ongoing inflammation. Likewise, lymphocytes, crucial to the adaptive immune system, are also elevated in MetS. This rise reflects a prolonged immune response to chronic low-grade inflammation, often associated with obesity and insulin resistance. Persistent inflammation in obese individuals disrupts normal immune function, resulting in continuous lymphocyte activation, which exacerbates insulin resistance, contributes to endothelial dysfunction, and accelerates atherosclerosis—all key factors in the progression of MetS [46 ,39 ,38] .

Insulin resistance, a condition that hampers insulin's effectiveness in promoting glucose uptake in adipose and muscle tissues and reducing glucose production in the liver, can lead to the accumulation of inflammatory markers, including total leukocytes [47-50]. This inflammation can also reduce the production of prostacyclin and nitric oxide, compromising endothelial integrity and function and leading to increased WBC counts and their subtypes, such as neutrophils and lymphocytes. Additionally, adipose tissue consistently expresses TNF-α, contributing to elevated leukocyte levels due to these proinflammatory cytokines [51].

On the other hand, the reduction of HDL-C in individuals with MetS is also attributed to several known factors. Chronic inflammation, characterized by increased pro-inflammatory cytokines such as TNF-α and IL-6, impairs HDL-C production and function, reducing its anti-inflammatory and antioxidant properties. Insulin resistance further contributes to dyslipidemia, decreasing HDL-

C production and raising triglyceride levels. Moreover, adipose tissue dysfunction, particularly in visceral fat, leads to higher secretion of inflammatory cytokines, which not only increase systemic inflammation but also reduce HDL-C synthesis and accelerate its clearance from circulation [52-54].

The clinical significance of NHR and LHR lies in their potential as accessible and cost-effective biomarkers for predicting MetS. These ratios, derived from routine blood tests, provide a practical and reliable approach for early diagnosis and risk stratification, particularly given gender-specific differences. Their integration into clinical practice facilitates the early identification of individuals at elevated risk, allowing for preventing complications such as CVD and diabetes. Additionally, NHR and LHR are valuable for monitoring the effectiveness of therapeutic interventions, reinforcing their role in both the prevention and management of MetS. Their ease of measurement, affordability, and applicability across diverse healthcare settings make them vital tools for improving patient outcomes through early detection and tailored treatment strategies, ultimately contributing to enhanced population health [38, 39] .

Strengths and Limitations

The research's strengths include its use of a large, population-based cohort from the Bandar-Kong Non-Communicable Diseases study, which provides robust data on a broad demographic within southern Iran. This strengthens the reliability of the findings and makes them more representative of the local population. Additionally, the study's focus on simple, accessible hematological markers (NHR and LHR) for predicting MetS is a practical contribution, as these markers are

easily measured in routine blood tests, making the findings potentially useful in standard clinical practice for early identification and monitoring of MetS risk. Moreover, the study's design allowed for a comprehensive analysis of associations between NHR and LHR and various MetS components, further supporting the value of these markers as diagnostic tools. The careful adjustment for potential confounders like age, gender, BMI, and physical activity levels adds to the study's rigor, helping isolate the specific impact of these hematological indices on MetS.

This study has several limitations that should be acknowledged. First, the cross-sectional design restricts the ability to establish causality between the hematological indices (NHR and LHR) and the development of MetS. Although associations were identified, causal relationships cannot be inferred from this type of study. Second, the study was conducted within a specific geographic region of southern Iran, which may limit the generalizability of the findings to other populations or ethnic groups. Differences in genetic, environmental, and lifestyle factors could influence the applicability of these results to broader or more diverse populations.

Third, while the study adjusted for several confounding factors such as age, gender, BMI, and smoking status, there may still be unmeasured confounders that could affect the associations observed. Furthermore, there was not information regarding lactating. Fourth, the study did not include other potential inflammatory biomarkers or cytokines that could provide a more comprehensive understanding of the inflammatory processes involved in MetS. Inclusion of markers such as high-sensitivity hs-CRP or IL-6 could have enriched the analysis. Lastly, the reliance on self-reported data for some variables, such as alcohol consumption, physical activity and smoking status, may introduce bias due to inaccurate or incomplete reporting. Objective measurements or longitudinal data could help mitigate this issue in future research.

Conclusion

In conclusion, the study's findings underscore the value of LHR and NHR as effective tools for identifying individuals at risk for MetS, particularly in resource-limited clinical settings where cost and accessibility are critical factors. The strong correlations between these hematological indices and key components of MetS highlight their potential as practical, cost-effective markers for early detection and intervention. This could play a significant role in managing and preventing MetS-related complications. The clinical relevance of this study lies in the practical application of LHR and NHR as accessible and affordable markers that can be integrated into routine patient care. By using these indices for regular assessments, especially in low-resource environments, healthcare providers can identify at-risk individuals early, allowing for timely interventions to prevent severe complications, such as CVD and T2DM. Implementing LHR and NHR as part of standard MetS screening protocols would thus enhance proactive management and contribute to better patient outcomes.

However, it is important to recognize that baseline differences and varied responses among different populations may influence the strength and nature of these associations. Therefore, future research, including meta-analyses, should aim to encompass a broader range of populations and include subgroup analyses and meta-regression. Such studies would enhance the understanding of how individual characteristics affect the relationship between LHR, NHR, and MetS, thereby refining the applicability and limitations of these markers in diverse clinical settings worldwide.

These advancements could further support the development of targeted intervention thresholds, enhancing the effectiveness of MetS management in diverse patient populations.

ithenticate_40084494.pdf

ORIGINALITY REPORT

14%

SIMILARITY INDEX

PRIMARY SOURCES

1	www.researchgate.net<br>Internet	65 words — 1%
2	thrc.hums.ac.ir<br>Internet	51 words — 1%
3	www.dovepress.com<br>Internet	47 words — 1%
4	www.frontiersin.org<br>Internet	45 words — 1%
5	www.ncbi.nlm.nih.gov<br>Internet	43 words — 1%
6	Sara Bahri, Masoumeh Kheirandish, Shideh Rafati, Azim Nejatizadeh et al. "Prevalence of Obesity and its Associated Factors Among the 35-70-Year-Old Population of Bandare-Kong: A Cross-sectional Survey (Findings of the Persian Cohort Study)", Disease and Diagnosis, 2022<br>Crossref	40 words — 1%
7	www.nature.com<br>Internet	29 words — < 1%
8	d.docksci.com<br>Internet	27 words — < 1%

9 Gordon Ferns. "Cause, consequence or coincidence: The relationship between psychiatric disease and metabolic syndrome", Translational Metabolic Syndrome Research, 2018 — 26 words — < 1%
Crossref

10 Ishwarlal Jialal, Ganesh Jialal, Beverley Adams-Huet, Neeraj Ramakrishnan. "Neutrophil and monocyte ratios to high-density lipoprotein-cholesterol and adiponectin as biomarkers of nascent metabolic syndrome", Hormone Molecular Biology and Clinical Investigation, 2020 — 22 words — < 1%
Crossref

11 James M. Rippe. "Lifestyle Nutrition - Eating for Good Health by Lowering the Risk of Chronic Diseases", CRC Press, 2024 — 22 words — < 1%
Publications

12 Jane Salier Eriksson, Björn Ekblom, Gunnar Andersson, Peter Wallin, Elin Ekblom-Bak. " Scaling VO max to body size differences to evaluate associations to CVD incidence and all-cause mortality risk ", BMJ Open Sport & Exercise Medicine, 2021 — 22 words — < 1%
Crossref

13 Xiaojiao Zeng, Guohong Liu, Yunbao Pan, Yirong Li. "Development and validation of immune inflammation–based index for predicting the clinical outcome in patients with nasopharyngeal carcinoma", Journal of Cellular and Molecular Medicine, 2020 — 21 words — < 1%
Crossref

14 link.springer.com — 21 words — < 1%
Internet

15 Di Zeng, Jiong Lu, Jinhong Chen. "Exploring the Correlation Between Gallstone Disease and — 19 words — < 1%

Sarcopenia Risk: Insights from a Cross-Sectional Analysis", Springer Science and Business Media LLC, 2024
Crossref Posted Content

16	conservancy.umn.edu<br>Internet	19 words — < 1%
17	worldwidescience.org<br>Internet	18 words — < 1%
18	Amin Ghanbarnejad, Masoumeh Kheirandish, Feysal Yousefzade, Arash Rahimi, Abnoos Azarbad, Azim Nejatizadeh, Mehdi Shahmoradi. "Metabolic syndrome severity score in the middle-aged and elderly Iranian population: A cross-sectional survey of Bandare-Kong Cohort Study (the findings of PERSIAN Cohort Study)", Frontiers in Public Health, 2023<br>Crossref	17 words — < 1%
19	www.mdpi.com<br>Internet	17 words — < 1%
20	lipidworld.biomedcentral.com<br>Internet	16 words — < 1%
21	"Sunday, 31 August 2008", European Heart Journal, 09/02/2008<br>Crossref	14 words — < 1%
22	George A. Bray. "Handbook of Obesity -- Volume 1 - Epidemiology, Etiology, and Physiopathology, Third Edition", CRC Press, 2019<br>Publications	13 words — < 1%
23	Hudson, Elizabeth L.. "Examination of the Effectiveness of Nutrition and Physical Activity Interventions on Behavior Change and Cardiovascular Risk	13 words — < 1%

Among Adolescent Students Participating in a School-Based Health Program in Michigan", Michigan State University, 2022
ProQuest

24	healthdocbox.com<br>Internet	13 words — < 1%
25	"JBS 2: Joint British Societies' guidelines on prevention of cardiovascular disease in clinical practice", Heart, 2005<br>Crossref	12 words — < 1%
26	clinmedjournals.org<br>Internet	12 words — < 1%
27	www.researchsquare.com<br>Internet	12 words — < 1%
28	Mehdi Shahmoradi, Ladan Hajiabdolrrasouli, Masoumeh Kheirandish, Shideh Rafati et al. "The Association Between Hematological Indices and Type 2 Diabetes Mellitus in Iranian Population", Shiraz E-Medical Journal, 2024<br>Crossref	11 words — < 1%
29	Noriyuki NAKANISHI, Mitsuru SATO, Kokoro SHIRAI, Kazue NAKAJIMA, Shigeki MURAKAMI, Toshio TAKATORIGE, Kenji SUZUKI, Kozo TATARA. "Associations between White Blood Cell Count and Features of the Metabolic Syndrome in Japanese Male Office Workers.", INDUSTRIAL HEALTH, 2002<br>Crossref	11 words — < 1%
30	www.rcompanion.org<br>Internet	11 words — < 1%

31	Jiahui Zhao, Qifan Zheng, Yue Ying, Shiyin Luo, Nan Liu, Liu Wang, Tong Xu, Aijia Jiang, Yaping Pan, Dongmei Zhang. "Association between high-density lipoprotein-related inflammation index and periodontitis: insights from NHANES 2009–2014", Lipids in Health and Disease, 2024<br>Crossref	10 words — < 1%
32	slideheaven.com<br>Internet	10 words — < 1%
33	wprim.whocc.org.cn<br>Internet	10 words — < 1%
34	www.huronregional.org<br>Internet	10 words — < 1%
35	www.sciencegate.app<br>Internet	10 words — < 1%
36	mail.pjms.org.pk<br>Internet	9 words — < 1%
37	Dana Dabelea, Georgeanna J. Klingensmith. "Epidemiology of Pediatric and Adolescent Diabetes", CRC Press, 2019<br>Publications	8 words — < 1%
38	Ingrid Kohlstadt. "Advancing Medicine with Food and Nutrients", CRC Press, 2019<br>Publications	8 words — < 1%
39	Parisa Hashemi Moghanjoughi, Sina Neshat, Abbas Rezaei, Kiyan Heshmat-Ghahdarijani. "Is the Neutrophil-to-lymphocyte ratio an exceptional indicator for metabolic syndrome disease and outcomes?", Endocrine Practice, 2021	8 words — < 1%

Crossref

40	Yonggang Ma, Fa Lin, Minghao Liu, Runting Li, Ke Wang, Donghai Wang, Xiaolin Chen, Chao Wang. "Relationship between De Ritis and clinical outcomes in patients with aneurysmal subarachnoid hemorrhage: Insights from the LongTEAM registry", Springer Science and Business Media LLC, 2024<br>Crossref Posted Content	8 words — < 1%
41	Zia, Anam. "Evaluation of the Relationship Between Medications and Falls Among Urban Community Dwellers in Malaysia", University of Malaya (Malaysia), 2023<br>ProQuest	8 words — < 1%
42	cardiab.biomedcentral.com<br>Internet	8 words — < 1%
43	Vlad Vunvulea, Ovidiu Aurelian Budișcă, Emil Marian Arbănași, Adrian Vasile Mureșan et al. "The Predictive Role of Systemic Inflammatory Markers in the Development of Acute Kidney Failure and Mortality in Patients with Abdominal Trauma", Journal of Personalized Medicine, 2022<br>Crossref	7 words — < 1%
44	Isaias Dichi, José Wander Breganó, Andréa Name Colado Simão, Rubens Cecchini. "Role of Oxidative Stress in Chronic Diseases", CRC Press, 2014<br>Publications	6 words — < 1%
45	Leo M.L. Nollet, Javed Ahamad. "Bioactive Compounds of Edible Oils and Fats - Health Benefits, Risks, and Analysis", CRC Press, 2024<br>Publications	6 words — < 1%

EXCLUDE QUOTES	ON	EXCLUDE SOURCES	OFF
EXCLUDE BIBLIOGRAPHY	ON	EXCLUDE MATCHES	OFF
